# Antiviral Activity of Biosynthesized Silver Nanoparticles from Pomegranate (*Punica granatum* L.) Peel Extract against Tobacco Mosaic Virus

**DOI:** 10.3390/plants12112103

**Published:** 2023-05-25

**Authors:** Abdulaziz A. Al-Askar, Dalia G. Aseel, Hamada El-Gendi, Sherien Sobhy, Marwa A. Samy, Esraa Hamdy, Sarah El-Messeiry, Said I. Behiry, Toufic Elbeaino, Ahmed Abdelkhalek

**Affiliations:** 1 Department of Botany and Microbiology, College of Science, King Saud University, P.O. Box 2455, Riyadh 11451, Saudi Arabia; aalaskara@ksu.edu.sa; 2 Plant Protection and Biomolecular Diagnosis Department, Arid Lands Cultivation Research Institute, City of Scientific Research and Technological Applications, Alexandria 21934, Egypt; daliagamil52@gmail.com (D.G.A.); sherienmorsey4@gmail.com (S.S.); smarwa201291@gmail.com (M.A.S.); esraah752@gmail.com (E.H.); 3 Bioprocess Development Department, Genetic Engineering and Biotechnology Research Institute, City of Scientific Research and Technological Applications, New Borg El-Arab City 21934, Egypt; elgendi1981@gmail.com; 4 Department of Genetics, Faculty of Agriculture, Alexandria University, Alexandria 21545, Egypt; sarah.elmesseiry@alexu.edu.eg; 5 Agricultural Botany Department, Faculty of Agriculture (Saba Basha), Alexandria University, Alexandria 21531, Egypt; said.behiry@alexu.edu.eg; 6 Istituto Agronomico Mediterraneo di Bari, Via Ceglie 9, 70010 Valenzano Bari, Italy

**Keywords:** tobacco mosaic virus, silver nanoparticles, antiviral activity, *Punica granatum* peel, antioxidant enzymes, polyphenolic genes, pathogenesis-related genes

## Abstract

Tobacco mosaic virus (TMV) is a major pathogen affecting tomato plants worldwide. The efficacy of silver nanoparticles (Ag-NPs) mediated by *Punica granatum* biowaste peel extract in mitigating the negative impact of TMV infection on tomato growth and oxidative stress was investigated through scanning electron microscopy (SEM), transmission electron microscopy (TEM), UV-Visible (UV-Vis) spectrophotometer, X-ray Diffraction (XRD), dynamic light scattering (DLS), zeta potential, energy-dispersive X-ray spectroscopy (EDX), and Fourier-transform infrared spectra (FTIR). Results of SEM analysis of green Ag-NPs revealed the presence of condensed spherical or round NPs with diameters ranging between 61 and 97 nm. TEM confirmed the SEM results and showed round-shaped Ag-NPs with an average size of 33.37 ± 12.7 nm. The elemental analysis (EDX) of prepared Ag-NPs revealed the presence of elemental Ag as a major peak (64.43%) at 3–3.5 KeV. The FTIR revealed several functional groups on the prepared Ag-NPs, for which three treatment strategies for Ag-NP applications were evaluated in the greenhouse study and compared to inoculated TMV and control plants: pre-infection treatment (TB), post-infection treatment (TA), and dual treatment (TD). The results showed that the TD strategy is the most effective in improving tomato growth and reducing viral replication, whereas all Ag-NP treatments (TB, TA, and TD) were found to significantly increase expression of the pathogenesis-related (PR) genes PR-1 and PR-2, as well as polyphenolic compounds, HQT, and C4H genes compared to control plants. In contrast, the flavonoid content of tomato plants was not affected by the viral infection, while the phenolic content was significantly reduced in the TMV group. Furthermore, TMV infection led to a significant increase in oxidative stress markers MDA and H_2_O_2_, as well as a reduction in the enzymatic activity of the antioxidants PPO, SOD, and POX. Our results clearly showed that the application of Ag-NPs on TMV-infected plants reduces virus accumulation, delays viral replication in all treatments, and greatly enhances the expression of the CHS gene involved in flavonoid biosynthesis. Overall, these findings suggest that treatment with Ag-NPs may be an effective strategy to mitigate the negative impact of TMV infection on tomato plants.

## 1. Introduction

An expanding global population, urbanization, and climate change are driving the need to increase crop yields and the quality of food production. In recent decades, the rate of agricultural production has been accelerated and improved in various ways, whereas plant diseases have remained significant constraints, hindering agricultural progress [1]. Plant biosecurity is seriously threatened by viral plant infections, which cause massive agricultural losses around the world [2]. Due to its numerous plant hosts (about 66 families with more than 900 plant species), the tobacco mosaic virus (TMV) is one of the most contagious plant diseases [3], with severe infection effects and stability outside host cells [4]. TMV is mechanically spread by touching infected plants, contaminated farming equipment, or infected seeds [5]. Even persistent viral particles can initiate the infection cycle after several years, attributed to high viral stability [6]. Hence, TMV was ranked first among the 10 most important viruses in terms of economic and scientific impact [7]. In general, two main approaches are applied for viral control: breeding resistant plant species or interfering with insect vectors of transmission [8,9]. The ability of TMV to evade plant resistance, in addition to the need for heavy insecticide application, greatly challenges the two approaches [2,9]. The application of hazardous agrochemicals to control viral infections is typically linked to decreased crop quality, serious health problems for crop consumers, and accumulation in waterbodies, which complicates environmental issues [9,10]. Hence, there is growing interest in the use of eco-friendly alternatives to harmful pesticides currently used for plant infection control to sustain agriculture and the environment [11].

Tomatoes are one of the most important and widely consumed vegetables in the *solanaceous* family, second only to potatoes [12,13]. Because of their high consumption rates, tomatoes are usually grown in greenhouses compared to field production [14,15], which increases their susceptibility to infection. TMV infection significantly affects the yield and quality of tomato crops [16]. Leaf mosaic and/or necrosis, in addition to leaf chlorosis, are further morphological indications of TMV infection in tomatoes [17]. Moreover, the infection may result in systemic alterations in the floral organs that postpone the ripening of the fruit, impair crop output, and cause crop loss [18]. Under abiotic and biotic stresses, plants usually respond through the activation of several mechanisms to alleviate the destructive effects of the ongoing challenge. This mechanism is usually regulated by signaling molecules and involves the activation of antioxidant power (enzymes and molecules) and pathogenesis-related genes [19].

In recent decades, nanoparticles (NPs) have attracted great attention for their diverse biological activities, ease of preparation, and stability under harsh application conditions [20,21]. NPs are usually privileged over their ancestor metals for their large surface-to-volume ratio and ability to accumulate several functional groups on their surface in corona-like structures, which significantly increase their application scope and largely enhance their stability [22,23]. Pomegranate-pericarp extract-mediated zinc oxide nanocomposites are effective as ROS scavengers and antibacterial agents [24]. The ability of NPs to interfere with plant viral infection and induce plant immune responses has been widely reported, with several promising impacts and lower toxicity to plants and the environment [25,26,27]. Among others, silver nanoparticles (Ag-NPs) have revealed significant antiviral activity against several plant infections. Pepper mild mottle virus was efficiently controlled by Ag-NPs in pepper seedlings [28], whereas Ahsan reported the successful application of *Pseudomonas fluorescens*-mediated Ag-NPs in controlling TMV in *Nicotiana glutinosa* plants [29]. Considering the economic importance of tomato crops and the promising antiviral activity of Ag-NPs, this study reports the effect of the application of these molecules on TMV-infected tomato plants as potential bio-stimulatory components with antiviral action rarely reported in the literature.

## 2. Results and Discussion

### 2.1. Pomegranate Peel Extract Mediated Ag-NPs Preparation

Green synthesis of nanoparticles has recently emerged as an eco-friendly approach for diverse nanoparticle preparation [30,31]. Hence, the Ag-NPs applied during this current work were prepared through pomegranate peel extract, as indicated by the dark-brownish color transformation of the precursor solution. Similarly, pomegranate peel extract was reported for the preparation of selenium and iron nanoparticles, which could be attributed to their contents of reducing enzymes and polyphenolic compounds [32,33].

### 2.2. Instrumental Characterization of Ag-NPs

**Figure 1:**
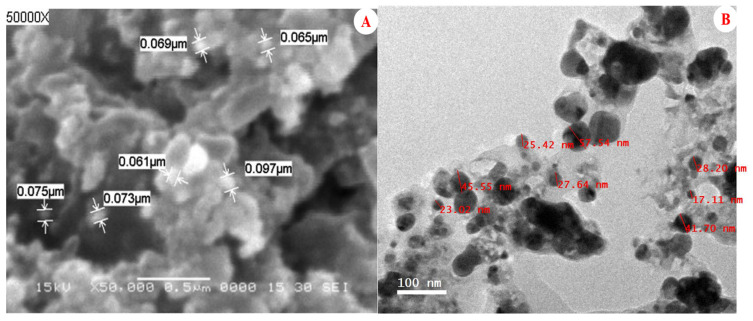
Morphological and particle characterization of the prepared Ag-NPs, illustrated by SEM analysis at 5000X (**A**) and TEM results (**B**).

SEM analysis (Figure 1A) indicated that the green synthesized Ag-NPs revealed condensed spherical to round-shaped NPs with different sizes from 61 to 97 nm, which could be attributed to the growth phase of the NPs. TEM analysis (Figure 1B) confirmed the SEM results and revealed round-shaped Ag-NPs with sizes ranging from 17.11 to 57.54 nm and an average size of 33.37 ± 12.7 nm, which is almost the same size as the Ag-NPs reported by Hawar et al. [34] and Kaliammal et al. [35]. TEM results indicated that the NPs surfaces are surrounded by a nonmetal layer, which could be attributed to organic materials from plant extract [36]. Hence, the larger sizes reported in the SEM results could be attributed to the interference of the organic capping with particle size measurements. Several studies reported the ability of NPs to accumulate organic material on their surfaces during the preparation process, especially when prepared using green approaches, which greatly influences the NPs’ activity, toxicity, and stability [33,37]. 

**Figure 2:**
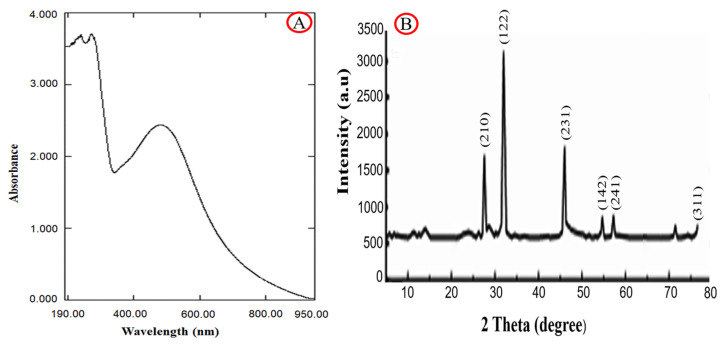
UV-VIS spectral analysis (**A**) and XRD pattern (**B**) of biosynthesized Ag-NPs.

The UV-Vis absorption spectra of the synthesized Ag-NPs were tested in the 190–950 nm range (Figure 2A). In most cases, Ag-NPs exhibit a surface plasmon resonance (SPR) band between 450 and 550 nm [38,39] as a result of the excitation of free electrons. Figure 1C shows that the SPR value of Ag-NPs was found to be 485 nm, which agrees with many previous studies [40,41,42]. XRD analysis confirmed the crystalline nature of Ag-NPs (Figure 2B). The diffraction pattern exhibited four distinct peaks at 2θ values of 32.34°, 46.13°, 54.17°, and 77.34°, which corresponded to (122), (231), (142), and (311), respectively (Figure 2B). These peaks are typical of the metallic face-centered cubic (fcc) phase of Ag and match with the database base of standard (JCPDS file no. 04-0783), which proves that the biosynthesized Ag-NPs were crystalline. Previous literature has reported identical values [43]. Additionally, it was observed that a limited number of peaks were not assigned, indicating the possibility of bio-organic phase crystallization on the surface of the synthesized Ag-NPs. Alternatively, this could be attributed to the involvement of phytoconstituents in the extract during the biosynthesis and stabilization processes of silver nanoparticles, as suggested by previous studies [44,45].

**Figure 3:**
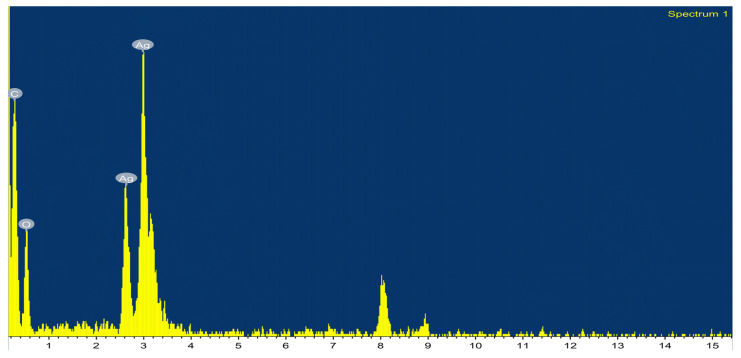
EDX examination of Ag-NPs produced by biosynthesis and reduced using pomegranate peel extract.

**Figure 4:**
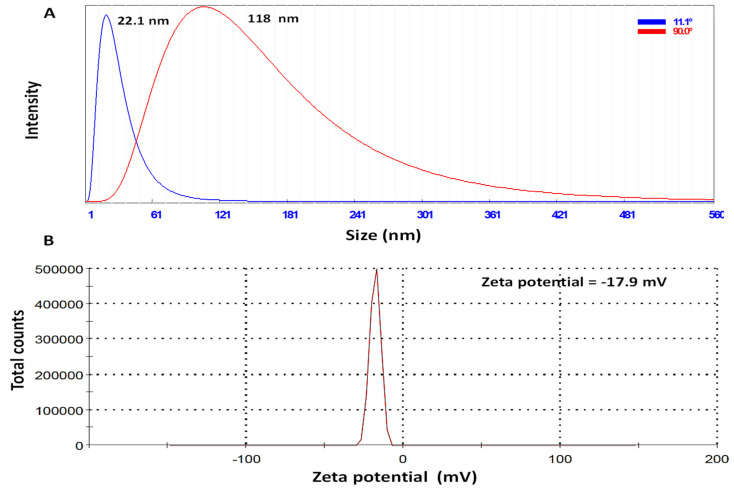
The DLS analysis of the particle size distribution of the prepared Ag-NPs through Zetasizer’s two angles 11° and 90° (**A**) and the particle’s surface charge (zeta potential) in (**B**).

On the other hand, the elemental analysis (EDS) of prepared Ag-NPs (Figure 3) revealed the presence of elemental Ag as a major peak (64.43%) at 3–3.5 KeV, which characterizes the surface plasmon resonance of Ag-NPs [46,47]. Additionally, carbon and oxygen were also detected in the NP sample, representing 25.4 and 10.17%, respectively. The presence of carbon and oxygen indicated the presence of organic material [46], which is in line with the TEM results. The technique of Dynamic Light Scattering (DLS) is commonly employed in the determination of the distribution of particle sizes present in a colloidal solution [48]. The particle size distribution of the green synthesized Ag-NPs was evaluated through the Zetasizer at two angles. The results (Figure 4A) asserted the formation of Ag particles in the nano-size range of 22.1 to 118 nm at 11° and 90°, respectively. The measured particle sizes are relatively higher than those reported through TM results (from 17.11 to 57.54 nm), which could be attributed to the tendency of the prepared particle to aggregate in the colloidal solution and/or polydisperse nature (non-homogeneous particle size) of the prepared particles, which is in line with other studies [25,49,50]. The zeta potential results (Figure 4B) revealed a negative surface charge of Ag-NPs of about −17.9 mV, which indicated the stability of the prepared particles in biological systems [51]. This negative charge of the prepared Ag-NPs could be a result of the adsorption of negative functional groups (OH-, CHO-, etc.) on the surface of Ag-NPs from the pomegranate peel extract [21,52,53,54].

**Figure 5:**
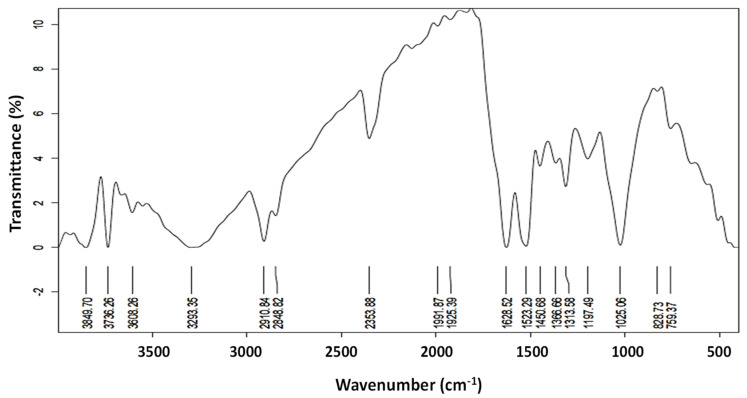
Fourier-transform infrared spectra of Ag-NPs produced via biosynthesis and reduced using pomegranate peel extract.

Furthermore, the function group analysis via Fourier-transform infrared (FTIR) (Figure 5) revealed several functional groups (18 peaks) on the prepared Ag-NPs. Major broadband at 3293.35 cm^−1^ was detected, which indicated the stretching vibration of the O-H group that belongs to alcohols and polyphenolic compounds and the -NH_2_ (amine) groups [36]. The peak at 2910.84 cm^−1^ indicated the stretching vibration of C-H (alkane) groups [31], while the peak at 2353.88 cm^−1^ confirmed the presence of C=O groups (aldehydes and ketones) in the prepared Ag-NPs. Additionally, the peak detected at 1628.52 cm^−1^ indicated the carbonyl groups’ (C=O) stretching vibration and the N-H bending of the amine group [36]. The two small bands at the 1450 and 1366 cm^−1^ indicated the stretching of the C-N and bending of NH (for the 1450 cm^−1^ peak) and the C-H of the aldehyde group (for the 1366 cm^−1^ peak), which confirm the existence of aliphatic groups of amid II [33]. The peaks around 1025.06- and 825 cm^−1^ indicated the C-O vibration, N-H, C-H, and C=CH_2_ stretching, respectively. These functional groups reduced Ag-NPs and enhanced the newly formed NPs stability, as reported in previous studies [31,33,55].

### 2.3. Plant Growth under Greenhouse Conditions

The growth of tomato plants was evaluated under greenhouse conditions after 19 days of TMV infection in all treatment groups. The results (Table 1) indicate a slight reduction (about 6%) in the fresh weight of TMV-challenged plants, as shown in the TMV group (7.97 ± 1.05 g), compared with the control plants (8.49 ± 0.55 g). Treatment with Ag-NPs enhanced the growth of TMV-challenged plants to normal levels, with a significant fresh weight in the TD groups (10.33 ± 1.19 g) representing an increase of about 21.7% compared with the control group, which asserts the potential of green synthesized Ag-NP application to improve tomato growth under TMV infection. Regarding shoot and root length, the results indicated a reduction in the shoot and root systems in the TMV group by about 10 and 30%, respectively, compared with the control plants. These results are in line with the fresh and dry weight results and indicate the direct impact of TMV infection on plant growth [16]. The prophylactic application of Ag-NPs (48 h before infection) revealed a significant impact by enhancing the shoot and root growth by 8 and 15%, respectively, compared with control plants. In the TD group, the results showed an 11% increase in the shoot system compared to control plants, with an approximately 15% increase in the root system (as in the TB group). Overall, the results indicated the importance of the two-dose treatment strategy not only to alleviate the viral impact on plant growth but also to efficiently improve tomato growth in the presence of TMV. 

### 2.4. Effect of Ag-NP Treatment on Oxidative Stress under TMV Challenge

**Figure 6:**
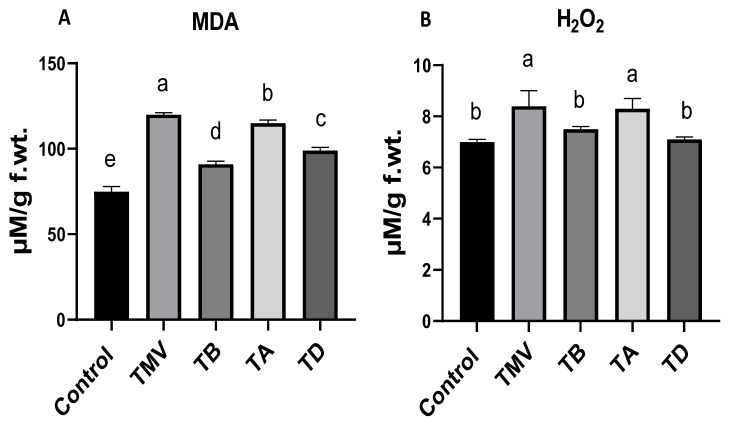
Oxidative stress markers: MDA (**A**) and H_2_O_2_ (**B**) on TMV-challenged tomato plants treated with Ag-NPs at 48 h before infection (TB), at 48 h after infection (TA), and dual treated at 48 h before and after infection (TD) compared with the healthy plant (Control) and untreated plants (TMV). The significance of the results is indicated in alphabetical letters above each column in ascending order.

Oxidative stress due to the overproduction of reactive oxygen species (ROS) is a major characteristic of viral infection, a condition that is related to the severity of infection [16,56]. Hence, two oxidative stress markers, MDA and H_2_O_2_, were evaluated to elucidate the oxidative stress in TMV-affected tomato plants in treated and untreated groups. The results (Figure 6A) indicated a sharp increase in the MDA titer in the TMV group (120 ± 1.2 µM/g f.wt.) compared with control plants (75 ± 2.9 µM/g f.wt.). The MDA elevation indicated peroxidation of polyunsaturated fatty acids in the cell, which takes place under oxidatively stressed conditions. The MDA molecule is very active, with the ability to interact with cellular DNA and proteins, and hence, impair normal cell functions [57]. In the treatment groups, prophylactic treatment (TB) was the most significant in MDA-level reduction (91 ± 1.8 µM/g f.wt.), followed by TD (99 ± 1.8 µM/g f.wt.). Though the MDA level was higher in the TA treatment by about 21% (compared to control plants), the Ag-NP application in the TA treatment reduced the MDA level by about 25% compared to the TMV group. 

Similarly, the accumulation and overproduction of H_2_O_2_ indicated uncontrolled oxidative stress in plant cells [56]. The results (Figure 6B) indicated an overproduction of H_2_O_2_ molecules (about 20%) in TMV groups (8.4 ± 0.6 µM/g f.wt.) compared with control plants (7.0 ± 0.1 µM/g f.wt.), which is consistent with Zhang et al. [8]. This accumulation could be attributed to the plant’s uncontrolled response to viral infection, which is usually accompanied by excessive production of ROS molecules [19]. The low level of ROS usually mediates signaling and activates the plant’s defense mechanisms against infection [58]. Treatment with Ag-NPs reduced the H_2_O_2_ levels in challenged plants in the TB (7.5 ± 0.1 µM/g f.wt.) and TD (7.1 ± 0.1 µM/g f.wt.) treatment groups. The inability of TA treatment to reduce H_2_O_2_ level (8.3 ± 0.4 µM/g f.wt.), in addition to the non-significant difference between TB and TD treatments, indicates that treatment time may be a limiting factor in controlling H_2_O_2_ accumulation and not treatment doses.

### 2.5. Effect of Ag-NP Treatment on Antioxidant Activity under TMV Challenge

**Figure 7:**
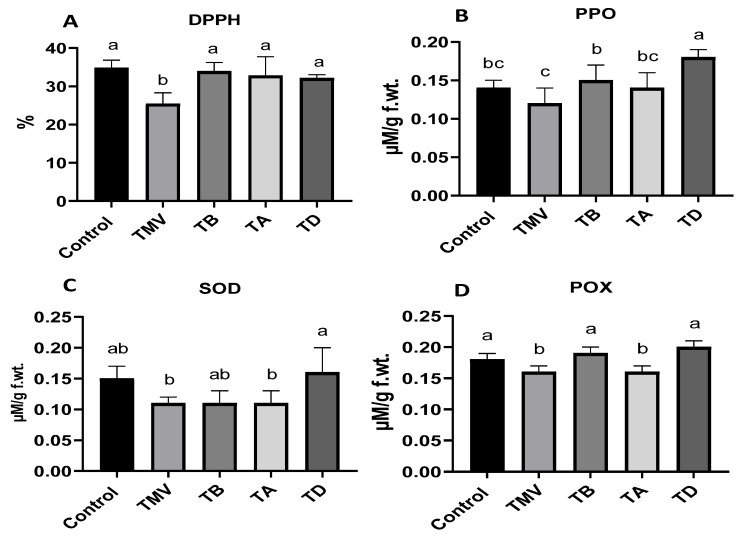
Antioxidant activity in terms of free radical scavenging as measured via DPPH (**A**) and three antioxidant enzymes: PPO (**B**), SOD (**C**), and POX activity (**D**) on tomato plants subjected to challenge by TMV treated with Ag-NPs at 48 h before infection (TB), 48 h after infection (TA), and dual treated at 48 h before and after infection (TD) compared with the healthy plant (Control) and untreated plants (TMV). The significance of the results is indicated in alphabetical letters above each column in ascending order.

The antioxidant potential of all groups was evaluated using leaf homogenate in terms of free radical scavenging activity and different antioxidant enzyme activities, including PPO, SOD, and POX. As shown in the results presented in Figure 7A, TMV infection reduced free radical scavenging activity by about 28%, as shown in the TMV group (25.4 ± 2.9%) compared with control plants. The hyperoxidative state promotes most viral infections and results from the uncontrolled response of plant cells to viral infection [59]. The Ag-NP application improved the free radical scavenging potential approximately to the control levels (control plants). Although there were non-significant differences in all treatment results, prophylactic application of Ag-NPs in TB treatment showed a slight increase in DPPH level (33.9 ± 2.3%) compared to the TA and TD groups (32.8 ± 4.9 and 32.1 ± 0.9%, respectively), which is in line with MDA and H_2_O_2_ results. 

The overproduction of antioxidant enzymes is an essential mechanism for plant cells to overcome viral infection and oxidative stress [60,61]. Hence, in this current study, three antioxidant enzyme levels were evaluated to elucidate the reason behind the antioxidant potential under the TMV challenge. The PPO enzyme was relatively inhibited under TMV infection (0.12 ± 0.02 µM/g f.wt.) compared to control plants (0.15 ± 0.02 µM/g f.wt.), as indicated in Figure 7B. The PPO mediated the plant protection against oxidative stress through phenolic compounds as final electron acceptors. Additionally, these phenolic compounds are usually deposited as lignin to enforce the cell walls and act as a physical barrier against any further infection [62]. Treatment with Ag-NPs enhanced the PPO activity approximately to control plant levels in TB and TA treatments (0.15 ± 0.02 and 0.14 ± 0.02 µM/g f.wt.), with a significant increase in TD treatment (0.18 ± 0.01 µM/g f.wt.). The PPO level in the TD group represents about 29 and 50% increases compared to control and TMV plants, respectively, which indicated the importance of a dual treatment strategy for PPO induction [16].

The SOD was also evaluated in all groups, and as indicated in the results (Figure 7C), the TMV infection reduced the enzyme level to 0.11 ± 0.01 µM/g f.wt., representing about a 27% reduction compared to control plants (0.15 ± 0.02 µM/g f.wt.). This enzyme catalyzed the incorporation of highly reactive superoxide species (O_2_^−^) with H_2_O inside the cell to give one molecule of H_2_O_2_, which was finally detoxified into molecular oxygen and water through glutathione peroxidases and/or catalases [16,63]. The reduction in SOD in the TMV group could elucidate the H_2_O_2_ accumulation detected in Figure 6B. Unexpectedly, the single treatment with Ag-NPs (TB or TA) was unable to enhance the SOD activity in TMV-challenged plants, whereas the two-dose treatment strategy (TD) significantly enhanced the enzyme titer (0.16 ± 0.04 µM/g f.wt.) by about 6 and 45% compared to control and TMV groups, respectively.

Regarding the POX level (Figure 7D), the TMV group revealed a slight reduction in the POX level (0.16 ± 0.01 µM/g f.wt.) by about 11% compared to control plants (0.18 ± 0.01 µM/g f.wt.). The role of POX in antioxidant activity involved the detoxification of ROS through organic material, with a final formation of lignin that precipitated in the cell wall and between cells to retard viral entry and internal movements within plant cells [63]. Some classes of the POX family were reported to interfere with viral replication and, hence, enhance plant resistance to such infections [64,65]. Application of Ag-NPs in TB and TD groups revealed significant enhancement in the POX level (0.19 ± 0.01 and 0.20 ± 0.01 µM/g f.wt.), even over the control plants’ levels. The TA treatment was unable to enhance the POX level, which indicated the importance of prophylactic Ag-NP application as an efficient strategy for activating POX production. 

### 2.6. Effect of Ag-NP Treatment on Polyphenolic Contents under TMV Challenge

**Figure 8:**
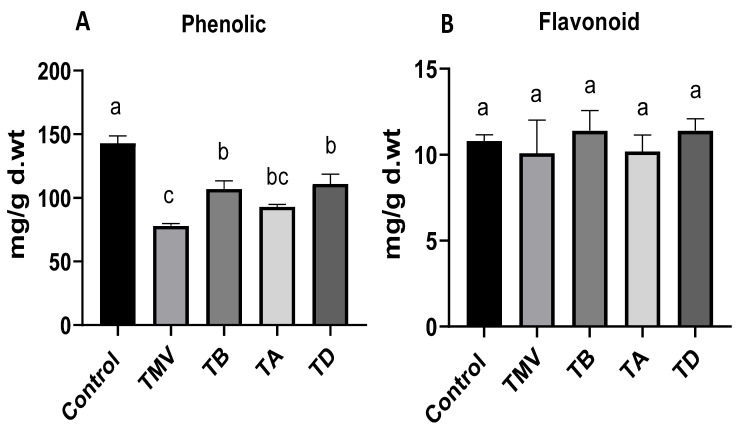
Polyphenolic compounds, including phenolic (**A**) and flavonoid contents (**B**), on tomato plants subject to TMV infection treated with Ag-NPs at 48 h before infection (TB), 48 h after infection (TA), and dual treated at 48 h before and after infection (TD) compared to healthy plants (Control) and untreated plants (TMV). The significance of the results is indicated in alphabetical letters above each column in ascending order.

The variation in the total polyphenolic contents (phenolic and flavonoids) in tomato plants under the TMV challenge was evaluated in all treatment groups. The phenolic content results (Figure 8A) indicated a significant reduction in TMV treatments (78 ± 1.7 mg/g d.wt.) by more than 45% from the control plants (143 ± 5.8 mg/g d.wt.). Phenolic compound accumulation plays a crucial role in plant resistance to various environmental and pathogenic challenges, as reported in various studies [66,67]. Hence, the downregulation of phenolic compound formation may be attributed to a viral strategy to evade plant resistance mechanisms. Treatment with Ag-NPs enhanced the phenolic content level, especially in the TB and TD treatment strategies to 93 ± 1.9 and 111 ± 7.6 mg/g d.wt., respectively. Though these results were lower than those in the control group, compared to the TMV treatment, the phenolic content was enhanced by 19% and 42% in the TB and TD treatments, respectively. On the other hand, the flavonoid contents (Figure 8B) were insignificantly affected by the viral infection (10.1 ± 1.92 7 mg/g d.wt.) compared to control plants (10.8 ± 0.36 mg/g d.wt.). Likewise, the Ag-NP application revealed insignificant effects on the flavonoid contents in treated groups, with a slight superiority for TB and TD strategies (about 11.436 mg/g d.wt.) compared to control plants (Figure 8B). Overall, the results indicated a minor potential for Ag-NP application to improve tomato resistance through the accumulation of polyphenolic compounds in the presence of TMV. However, the prophylactic application of Ag-NPs before the viral infection is slightly more effective in mitigating viral effects on polyphenolic content reduction than the other two strategies (TA and TD). 

### 2.7. Effect of Ag-NP Treatment on TMV Particle Accumulation and Pathogenesis-Related (PR) Gene Expression 

**Figure 9:**
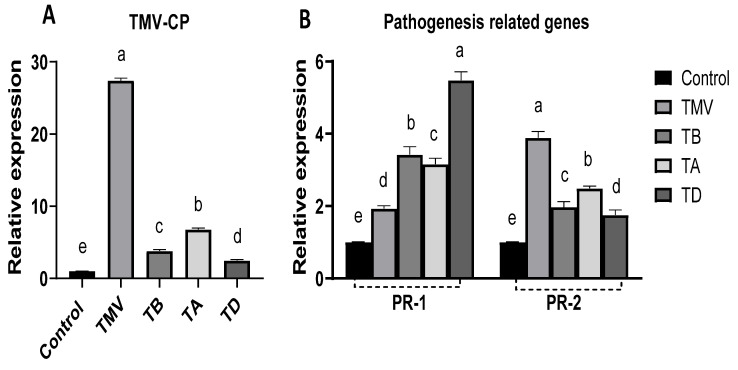
Accumulation of TMV particles (**A**), and expression of pathogenesis genes (**B**) including PR-1 and PR-2 on under TMV-challenged tomato plants treated with Ag-NPs at 48 h before infection (TB), 48 h after infection (TA), and dual treated at 48 h before and after infection (TD) compared to healthy plant (Control) and non-treated plants (TMV). The significance of the results is indicated in alphabetical letters above each column in ascending order.

The accumulation of viral particles within host cells as an intracellular infectious agent allows for the evaluation of infection severity [68]. Here, the amount of TMC-CP was measured in all treatments using qRT-PCR. This showed that the amount of TMV-CP was 27.42 times higher in TMV-treated plants than in control plants (Figure 9A). This accumulation is in line with the infection, symptoms, and growth parameters in the TMV group [8]. Application of Ag-NPs significantly reduced the TMV-CP accumulation (compared to TMV treatment) and, hence, retarded the viral replication as apparent in all treatments to 3.78-, 6.79-, and 2.48-fold increases in the TB, TA, and TD groups, respectively, compared to control plants. The results indicated a maximum TMV-CP reduction in the TD strategy of about 91% from TMV treatment, which asserted the efficiency of two doses of Ag-NPs for controlling TMV infection in tomatoes, with one of them used as a prophylactic application before infection. The data presented in this study provide evidence to support the hypothesis that Ag-NPs possess strong antiviral properties. The application of Ag-NPs to tomato plants resulted in a reduction in disease severity and concentration levels of ToMV and PVY within plant tissues, as reported in a previous study [69]. Moreover, the administration of Ag-NPs subsequent to 24 h of virus inoculation resulted in a reduction in virus concentration and infection rate, as reported in a previous study [70,71]. Upon entering plant cells, Ag-NPs initiate antiviral activity by interfering with cellular components or viral vectors, thereby preventing viral replication. This is achieved through the activation of DNA or RNA mechanisms [72]. Moreover, it has been demonstrated that Ag-NPs possess the ability to attach to the viral genome, thereby impeding polymerase activity and hindering the replication of the virus [73].

The activation of PR genes underlying the plant resistance mechanism is ongoing [19]. Among them, the PR-1 gene overexpression indicated the activation of systemic acquired resistance (SAR), which is usually regulated and mediated through salicylic acid [25]. The results (Figure 9B) revealed induction of PR-1 gene expression in the TMV group (about 93%) compared to control plants, which could be attributed to initial plant resistance to viral infection. Treatment with Ag-NPs greatly enhanced the PR-1 gene expression to more than 3-fold in TB and TB treatments, with maximum induction in the TD group of about 5.48-fold compared to control plants. These results asserted the ability of Ag-NPs to activate SAR pathways as the main plant resistance strategy in tomato plants under TMV challenge, which is in line with previously reported studies that reported Ag-NPs upregulating PR-1 even in viral infection [25,71]. Additionally, SAR activation through Ag-NPs is dose-dependent and requires a two-step application strategy for maximum efficiency.

On the other hand, the PR-2 gene regulated intracellular cell-to-cell movement through β-1,3-glucanase activity, which mediated callose hydrolyzing activity [74,75]. Hence, overexpression of the PR-2 gene is believed to facilitate intercellular plant viral spreading to adjacent cells. In this current study, the PR-2 evaluation results (Figure 9B) indicated significant induction of the gene expression related to viral infection as indicated in the TMV group (3.9-folds) compared to control plants, which could be attributed to the viral tendency to enhance its internal movements [76] and is in line with other studies [25,77]. In treated groups, the PR-2 expression level was significantly reduced compared to TMV groups, especially in TB (50%) and TD (55%) treatments. Though PR-2 gene expression in the two treatments (TB and TD) remains higher than in control plants, the results assert the ability of Ag-NPs to mitigate viral transmission by interfering with β-1,3-glucanase activity [25].

### 2.8. Effect of Ag-NP Treatment on Polyphenolic Gene Expression under TMV Challenge

**Figure 10:**
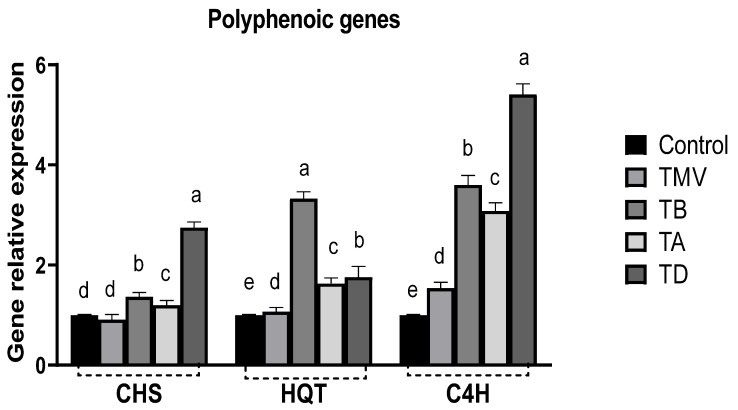
The relative expression of three polyphenolic genes, including CHS, HQT, and C4H, on tomato plants under TMV challenge treated with Ag-NPs at 48 h before infection (TB), 48 h after infection (TA), and dual treated at 48 h before and after infection (TD) compared to healthy plant (Control) and non-treated plants (TMV). The result significance was indicated in alphabetical letters above each column in ascending order.

Polyphenolic compounds are the main components of plant cells for diverse biological activity under abiotic and biotic stress [78,79]. These compounds are directly or indirectly involved in the scavenging of ROS to alleviate oxidative stress or in plant cell wall enforcement against biotic stresses [8,80]. Hence, three polyphenolic synthesis genes were evaluated in all treatment groups. The results (Figure 10) indicated a slight reduction in CHS gene expression in the TMV group (by about 10%) compared to control plants. The CHS (Chalcone synthase) catalyzes the synthesis of flavonoid precursors and a key intermediate called naringenin chalcones from p-coumaroyl CoA, hence playing a major role in flavonoid synthesis [81]. Treatment with Ag-NPs enhanced the CHS gene expression in TB (1.37-folds) and TA treatments (1.2-folds), with maximum relative gene expression in TD treatment (2.75-folds) compared to control plants. 

The HQT gene expression revealed a significant variation in viral infection, as indicated in the TMV treatment compared to control plants (Figure 10). The prophylactic application of Ag-NPs significantly enhanced the HQT gene expression up to 3.33-folds compared to control plants. This gene encodes an essential transferase enzyme that is involved in chlorogenic acid biosynthesis [82]. Several studies reported the essential role of chlorogenic acid as an antioxidant molecule in addition to various pathogen resistance mechanisms [83,84]. Though the TA and TD treatments also enhanced the HQT gene expression 1.63- and 1.76-fold compared to control plants, the results asserted the importance of prophylactic application of Ag-NPs for maximum HQT gene expression, which is in line with previously observed results [85] and could be attributed to the accumulation of the phenolic molecules and the triggering of SAR for viral infection [71]. Furthermore, the C4H gene encodes cinnamate 4-hydroxylase, which is a strategic enzyme for catechin biosynthesis. Several pathways of plants’ flavonoid synthesis rely on catechin as an intermediate [86]. Viral infection enhanced the C4H gene expression 1.54-fold compared to control plants, which could be attributed to the initial resistant response of tomato plants to TMV infection. All Ag-NP treatment groups revealed significant increases in C4H gene expression by more than three-fold in TB and TA treatments (Figure 10). TD treatment revealed C4H gene expression accounting for about 5.41-folds compared to control plants, which asserted the direct involvement of the C4H gene in alleviating the infection consequences in stressed plants [85,87,88].

## 3. Materials and Methods

### 3.1. Green Synthesis of Ag-NPs through Pomegranate Peel Extract 

The green synthesis of the Ag-NPs was carried out from AgNO_3_ using pomegranate (*Punica granatum* L.) peel extract. Initially, the plant extract was prepared by mixing 10 g of pomegranate peel with 100 mL of phosphate buffer at pH 7.0. After 2 h of shaking at 50 °C, the mixture was precipitated via centrifugation (10,000× *g* for 10 min), and 50 mL of clear supernatant was added to 50 mL of AgNO_3_ solution (1 mM, freshly prepared) and incubated under shaking at dark conditions. The development of a dark brown color indicated the AgNO_3_ reduction and formation of Ag-NPs, which were separated through centrifugation at 6000× *g* for 10 min. The precipitated Ag-NPs were washed several times with distillate water and absolute ethanol to ensure purity, then dried at 60 °C for 24 h before any application.

### 3.2. Instrumental Characterization of the Prepared Ag-NPs 

The green synthesized Ag-NPs were characterized through different instrumental techniques, as follows: the particles’ surface morphology was evaluated via scanning electron microscopy (SEM) operated at an acceleration voltage of 15 KV and magnifications of 5000X using a JSM-6360 LA microscope (Tokyo, Japan). X-ray diffraction (XRD) patterns were performed using an XRD-7000 (Shimadzu, Kyoto, Japan) diffractometer equipped with a CuK radiation beam (λ = 0.154060 nm), set to 30 KV and 30 mA, and collecting data at 10–80° in 2θ. The elemental analysis of the prepared Ag-NPs was conducted with the energy-dispersive X-ray spectroscopy (EDS) unit of the TEM microscope. Transmission electron microscopy (TEM) was used to elucidate the particle’s shape and size using the JSM-6360 microscope (JEOL, Tokyo, Japan). The particle size distribution determined via dynamic light scattering (DLS) and surface charge (zeta potential) in colloidal solutions was evaluated through the Zetasizer (ZS, Malvern, Germany). Additionally, the surface functional groups of the prepared nanoparticles were evaluated through Fourier-transform infrared spectroscopy (FTIR) using the KBr-disc approach in a range of 400–4000 cm^−1^. 

### 3.3. TMV Source

The applied TMV strain was isolated in our laboratory from severely infected tomato plants and identified, and its sequence deposited in GenBank under the accession number MG264131 [89]. The isolated virus was maintained in tobacco plants for further applications.

### 3.4. Tomato Source and Propagation under Greenhouse Conditions

The viral-free tomato seeds (*Solanum lycopersicum* L.) used in our experiments were obtained from the Agriculture Research Center (Egypt). Tomato plants were cultivated in round plastic pots (30 × 29.9 × 27.3 cm). Pots were provided with a mixture of sand and clay (4 kg/pot) in a final ratio of 1:1. To ensure sterility, the prepared soil was previously sterilized through autoclaving. All pots were incubated under insect-free greenhouse conditions at a temperature of 28 °C/16 °C (day/night) with a relative humidity of 70%. After 28 days, the growing tomato seeds were transplanted into new pots and cultivated under the same conditions for another week. For TMV infection (infected groups), the tomato plant leaves were mechanically infected with 1 mL of viral solution, according to Hafez et al. [90]. 

### 3.5. Ag-NP Application for TMV-Controlling under Greenhouse Cultivations

In order to evaluate the effect of Ag-NP application for TMV control, greenhouse-grown tomato plants were allocated into five groups. Each group (treatment) included five replicated pots with five plants per pot. The five treatments included healthy group or control plants as negative control (Control group), TMV-infected plants as a positive control (TMV group), TMV-infected plants previously treated with Ag-NPs 48 h before viral infection (TB group), TMV-infected plants latterly treated with Ag-NPs 48 h after viral infection (TA group), and TMV-infected plants dual treated with Ag-NPs 48 h before and 48 h after viral infection (TD group). For Ag-NP application, each entire plant was foliar sprayed with 5 mL of Ag-NPs (100 g/mL) using a handheld pressure sprayer to cover all plant leaves. All plants were cultivated under greenhouse conditions as described previously [91] and monitored for symptoms and developments daily. After 19 days post-TMV-inoculation (19 dpi), samples were collected from each group (3 upper true leaves or plants) for different analyses. Furthermore, the plant’s shoot and root systems were harvested and weighed (fresh weight and dry weight) as an indication of plant growth under TMV-challenge and Ag-NP treatments. The shoot and root system lengths (cm/plant) were also evaluated in all treatment groups.

### 3.6. Effect of Ag-NP Treatment on Oxidative Stress under TMV Challenge

Oxidative stress in tomato plants under TMV challenge was elucidated in treated and untreated groups by assessing the levels of two oxidative stress markers, namely malondialdehyde (MDA) and hydrogen peroxide (H_2_O_2_). First, the plant leaves (5 g) were homogenized in 25 mL of trichloroacetic acid (TCA, 0.1% *w*/*v*) solution, then precipitated with centrifugation (13,000× *g* for 20 min) to obtain a clear leaf homogenate. The MDA level was evaluated through the thiobarbituric acid (TBA) approach by mixing clear leaf homogenate (300 μL) with 1 mL of TBA solution (prepared as 0.5% TBA in 20% TCA). The mixtures were incubated at 95 °C for 30 min, then directly immersed in ice to terminate the reaction [92]. The developed color was measured at 600 nm, indicating the MDA concentration (µM/g of fresh weight). On the other hand, the H_2_O_2_ was evaluated in all treatments through the potassium iodide (KI) approach [93] by mixing 2 mL of 1 M KI (prepared in 10 mM phosphate buffer, pH 7.0) with 1 mL of clear leaf homogenate at room temperature for 20 min. The H_2_O_2_ levels (µM/g of fresh weight) were deducted from the extinction coefficient of H_2_O_2_ (0.28 µmol/cm) after measuring the reaction at 390 nm.

### 3.7. Effect of Ag-NP Treatment on Antioxidant Activity under TMV Challenge

The antioxidant potential in all groups was evaluated using leaf homogenate in terms of free radical scavenging activity and different antioxidant enzyme titers. The leaf homogenate from each group was prepared by mixing 10 g of plant leaves in 50 mM phosphate buffer (pH 7.0) in a high-speed blender for 10 min. The mixture was then centrifuged at 13.000× *g* for 10 min, and clear leaf homogenate was applied in the following experiments.

#### 3.7.1. The Free Radical Scavenging Activity Screening

The ability to quench free radicals and reactive oxygen species was evaluated in all treatment groups as an indication of the antioxidant potential. In this regard, 2,2-Diphenyl-1-picrylhydrazyl (DPPH) was added to the plant extract, and the reduction in the reaction color was measured at 517 nm [62]. The reaction mixture included 2 mL of 0.05 M DPPH (in methanol) and 100 μL of leaf homogenate. The reaction was incubated at 30 °C for 30 min, and the reduction in the absorbance was compared to the control prepared with 100 μL of phosphate buffer (100%).

#### 3.7.2. Evaluation of Polyphenol Oxidase Activity

The quinone method was applied to evaluate the polyphenol oxidase (PPO) activity in all treatment groups according to Cho and Ahn [94] as follows: 500 µL of leaf homogenate was incubated with 1 mL of 50 mM quinone prepared in 100 mM Tris-HCl buffer (pH 6.0) at 25 °C for 10 min. Each 0.001 increase in the reaction absorbance represents one unit (U/min) of PPO activity at 420 nm.

#### 3.7.3. Evaluation of Superoxide Dismutase Activity

The photoreduction inhibition approach was applied to determine the superoxide dismutase (SOD) activity using nitroblue tetrazolium (NBT) salts as adapted from Beauchamp and Fridovich [95] as follows: a reaction mixture of 50 µM NBT, 50 mM sodium carbonate, 12 mM L-methionine, 0.1 mM EDTA, and 10 µM riboflavin was added to 100 µL of leaf homogenate. After adjusting the reaction volume to 3 mL with phosphate buffer (50 mM and pH 7.0), the photochemical reduction was initiated through direct illumination of the reaction mixture for 15 min using fluorescent lamps. The reaction was then incubated in dark conditions and measured at 560 nm. One unit of SOD activity (µmol/g of fresh weight) represents a 50% reduction in the reaction color compared to the homogenate-free reaction as a control. 

#### 3.7.4. Evaluation of Peroxidase Activity

The guaiacol oxidation in the presence of H_2_O_2_ was applied to evaluate the peroxidase activity (POX) in all treatment groups according to Angelini et al. [96] as follows: 500 µL of guaiacol (5 mM) and 120 µL of H_2_O_2_ (1 mM) were added to 80 µL of leaf homogenate. The reaction volume was adjusted with phosphate buffer (100 mM, pH 7.0) to 1200 µL and incubated at 30 °C. After 10 min, the absorbance was measured at 480 nm, whereas POX activity was deducted from the guaiacol extinction coefficient (ε = 26,600 M^−1^ cm^−1^). 

### 3.8. Effect of Ag-NP Treatment on Polyphenolic Contents under TMV Challenge

The variation in the total polyphenolic contents (phenolic and flavonoids) in tomato plants under the TMV challenge was evaluated in all treatment groups. First, the Folin–Ciocalteau approach was applied to evaluate phenolic contents as follows: 1 g of air-dried leaves was grounded in 50 mL of 80% methanol, then precipitated to form a clear methanol extract. Then, 200 μL of the methanol extract was added to 1 mL of Folin–Ciocalteau reagent for 5 min. Afterward, the final reaction volume was adjusted to 2.5 mL using a Na_2_CO_3_ solution (7.5% *w*/*v*) under vigorous shaking. After 60 min of incubation in dark conditions, the phenolic content was measured at 750 nm and deducted from a standard curve of gallic acid [97]. On the other hand, the total flavonoid contents were evaluated through a modified aluminum chloride (AlCl_3_) approach as follows: 500 μL of phosphate-buffered leaf extract (pH 7.0) was incubated with a reaction mixture (4.5 mL) of potassium acetate (100 μL, 1 M), methanol (1500 μL), and AlCl_3_ (100 μL, 10% *w*/*v*) for 30 min at 25 °C. Afterward, the reaction absorbance at 415 nm was used to deduce the total flavonoid using a standard curve of quercetin [98]. 

### 3.9. Effect of Ag-NP Treatment on Polyphenolic and Pathogenesis-Related (PR) Genes Expression under TMV Challenge

The variation in the expression levels of some polyphenols and PR genes, as well as TMV-coat protein (TMV-CP), was evaluated in all treatments through quantitative RT-PCR (qRT-PCR) compared to control plants. In this regard, the expression of three polyphenolic genes, namely CHS, HQT, and C4H, in addition to two PR genes, PR-1 and PR-2, was evaluated in all treatments using the cDNA as a template, as mentioned before [16,99]. Briefly, for cDNA synthesis, the total RNA of each treatment was first extracted from tomato leaves (3 leaves/plant in 5 replicates) at 19 dpi using RNeasy plant mini kit (QIAGEN, Germany). The cDNA was synthesized from extract DNase-treated RNA (1 μg) via a reverse transcription reaction according to previously reported [99] and stored at −20 °C for further applications. The synthesized cDNA in each treatment was applied as a template for different gene expression through real-time PCR equipment (Rotor-Gene 6000, QIAGEN, Germantown, MD, USA) using the SYBR Green Mix (Thermo, Foster, CA, USA) and a pair of specific primers (Table 2), as mentioned earlier [99]. The expression of the β-actin housekeeping gene was used to normalize the expression of genes under study, whereas the genes’ relative expression was calculated according to the 2^−ΔΔCT^ method [100]. 

### 3.10. Data Analysis

The Costat software was utilized to analyze the relative transcription values of each treatment. The Tukey post hoc test (HSD) was employed to calculate the disparities between the means at a significance level of *p* ≤ 0.05. Transcription values that exceed 1 indicate an elevation in gene transcription levels, which is referred to as up-regulation. Conversely, transcription values that are less than 1 indicate a decrease in transcriptional levels, which is known as down-regulation.

## 4. Conclusions

This study explored the efficacy of silver nanoparticles (Ag-NPs) mediated by *Punica granatum* biowaste peel extract in mitigating the negative impact of TMV infection on tomato plants. Moreover, this study characterized the Ag-NPs using different analytical techniques, such as SEM, DLS, TEM, EDX, FTIR, and zeta potential distribution. The results showed that treatment with Ag-NPs, particularly the dual-treatment strategy, significantly improved tomato growth, reduced viral replication, increased the expression of pathogenesis-related (PR) genes, and antioxidant enzymes, and restored flavonoid biosynthesis. Taking together all these outcomes, this study suggests that treatment with Ag-NPs could be a promising strategy to control TMV infection in tomato plants, which is a strategy to be extended to other viruses and that can serve for better management of viral diseases of many crops.

## Figures and Tables

**Table 1:** Tomato growth parameters in terms of fresh and dry weight (g) with shoot and root length (cm) on TMV-infested tomato plants treated with Ag-NPs at 48 h before infection (TB), 48 h after infection (TA), and dual treated at 48 h before and after infection (TD) compared to healthy plant (Control) and non-treated plants (TMV).

Treatment	Fresh Weight (g)	Dry Weight (g)	Shoot Length (cm)	Root Length (cm)
Control	8.49 ± 0.55 c	2.31 ± 0.42 d	33.00 ± 2.94 d	11.50 ± 3.00 c
TMV	7.97 ± 1.05 d	2.21 ± 0.22 e	30.00 ± 3.55 e	8.00 ± 3.61 d
TB	8.64 ± 1.77 b	2.38 ± 0.30 b	35.60 ± 3.64 b	13.20 ± 2.02 a
TA	8.71 ± 1.12 b	2.33 ± 0.24 c	33.50 ± 2.65 c	12.00 ± 2.97 b
TD	10.33 ± 1.19 a	2.50 ± 0.59 a	36.60 ± 3.51 a	13.20 ± 2.68 a

**Table 2:** The forward and reverse nucleotide sequences of the applied primes in this current study amplify the polyphenolic genes (CHS, HQT, and C4H), pathogenesis-related genes (PR-1 and PR-2), and TMV-coat protein (TMV-CP).

Primers	Abbreviation	Nucleotide Sequence (5′ to 3′)	References
Tobacco mosaic virus-coat protein	TMV-CP	Forward: CGACTGCCGAAACGTTAGA	[101]
		Reverse: AAGTTGCAGGACCAGAGGT	
Pathogenesis related protein-1	PR-1	Forward: GTTCCTCCTTGCCACCTTC	[102]
		Reverse: TATGCACCCCCAGCATAGTT	
Endoglucanase	PR-2	Forward: ATAGCCGTTGGAAACGAAG	
		Reverse: CAACTTGCCATCACATTCTG	
Chalcone Synthase	CHS	Forward: CACCGTGGAGGAGTATCGTAAGGC	[103]
		Reverse: TGATCAACACAGTTGGAAGGCG	
Hydroxycinnamoyl Co A: quinatehydroxycinnamoyl transferase	HQT	Forward: CCCAATGGCTGGAAGATTAGCTA	
		Reverse: ATGAATCACTTTCAGCCTCAACAA	
Cinnamic acid 4-hydroxylase	C4H	Forward: CCCAGTTTTTGGAAATTGGCTTCA	[85]
		Reverse: GCCCCATTCTAAGCAAGAGAACATC	
β-actin	β-actin	Forward: GGCATACAAAGACAGGACAGCCT	[104]
		Reverse: CTCAATCCCAAGGCCAACAGAGA	

## Data Availability

Experimental data supporting the findings of this study are available from the corresponding authors upon request.
